# Reinforcement and Maintenance of Human Resources for Health Systems during Long-Term Crises: A Systematic Review of Systematic Reviews

**DOI:** 10.1155/2021/9613443

**Published:** 2021-10-31

**Authors:** Amir Mohammad Salehi, Salman Khazaei, Motahareh Masumi, Farnaz Shavandi, Mostafa Kavand, Ensiyeh Jenabi, Mahnaz Khatiban

**Affiliations:** ^1^Student Research Committee, Hamadan University of Medical Sciences, School of Medicine, Hamadan, Iran; ^2^Research Center for Health Sciences, Hamadan University of Medical Sciences, Hamadan, Iran; ^3^Student Research Committee, Hamadan University of Medical Sciences, School of Public Health, Hamadan, Iran; ^4^Student Research Committee, Hamadan University of Medical Sciences, School of Nursing and Midwifery, Hamadan, Iran; ^5^Autism Spectrum Disorders Research Center, Hamadan University of Medical Sciences, Hamadan, Iran; ^6^Mother and Child Care Research Center, Department of Ethics Education in Medical Sciences, Department of Medical-Surgical Nursing, School of Nursing and Midwifery, Hamadan University of Medical Sciences, Hamadan, Iran

## Abstract

**Background:**

Human resources are one of the most critical organizational resources, the reinforcement and maintenance of whom require much energy in health organizations, particularly in long-term crises. Many methods have been suggested in this regard; however, there is a need for their integration and clarification.

**Methods:**

We systematically searched the international databases, including PubMed, Scopus, and Web of Science, from 2003 to April 2021by using some relevant keywords. The quality of the included studies was assessed using the AMSTAR checklist.

**Results:**

The search resulted in 1613 papers, among which there were 16 systematic reviews. The studies addressed a wide range of problems and solutions. Twelve items and four items were classified with moderate quality (AMSTER score 5–8) and high quality (AMSTER score 9–11), respectively. Half of the studies (*n* = 8) dealt with mental and psychological problems resulting from crises as the most important factor in the decline of health system staff's durability in organizations. They also provided different solutions such as mental health counselling during and after the crisis, flexible work schedule, promoted trust in the organization, support of staff's family, and enhanced awareness to support employees. And the other articles addressed managerial problems as the most critical factor in the decline of health system staff's durability in organizations and proposed solutions such as suitable planning before, during, and after the crisis and the use of material and spiritual incentives to increase the employees' motivation and organizational resilience to maintain the staff.

**Conclusion:**

In the present review study, three dimensions (namely, resilience, motivation-hygiene measures, and development of manager's soft skills) are considered as the main factors reinforcing and maintaining human resources in the health systems in long-term crises and disasters.

## 1. Introduction

The rate of crises and the number of their adverse consequences are increasing worldwide, which usually occur fast and suddenly [1]. According to Kristine Qureshi's report in 2005, the collected data on the sudden prevalence of severe acute respiratory syndrome (SARS) indicate that, in these crises, healthcare staff might be reluctant to work [2, 3]. Among the variety of crises, the nurses' minimum rate of reluctance to work (48%) was related to the SARS period [4]. Their reluctance could negatively influence the healthcare system's potentials to meet the needs of a sudden increase in the number of patients under such a condition [5, 6]. On the other hand, healthcare staff, in particular nurses, who make up the largest number of healthcare providers and play the pivotal role in crises response and care, are required to have sufficient knowledge, skills, competencies, and adequate preparation to respond to crisis [1, 7]. In this regard, the findings reported by Said and Chiang indicate that nurses should focus on psychological preparedness to develop and create better educational plans to reinforce knowledge and care abilities required in crises [1].

Modern challenges have been diverse and include a complicated range of issues such as climate changes, severe economic downturns, and political instabilities. In this interconnected world, these main issues could be a sudden threat to organizations' life and survival. That is why these kinds of challenges encourage organizations to stay adaptable and responsive while organizing and managing their staff [8]. Under such conditions, resilience makes systems adapt to emerging challenges and let organizations continue their activities in the new condition [9]. However, organizations have faced a huge challenge with the COVID-19 outbreak, which makes them manage their employees using different technical, physical, and social-psychological techniques never experienced before [8].

In a crisis, one of the critical duties of human resources (HR) management is maintaining employees' health while preserving their morals and loyalty and encouraging them to continue their activities in line with the organization's goals [10]. COVID-19 has created a challenging condition, particularly for the HR management since the HR department is one of the most pivotal parts of modern organizations, the role of which becomes even more highlighted during crises and in the crisis management [5, 8]. In this situation, many employees have been encouraged to work in their homes, and their managers monitor tasks remotely; however, health organizations, especially those with emergency personnel and healthcare specialists, need their staff in their workplace [2]. Under this condition, human resource managers should help the staff deal with changes occurring in the workplace and social environment and adapt themselves to these changes [8] since it affects how personnel are supposed to make decisions. Moreover, plans, policies, and organizational decisions should be made based on the best available evidence [3]. Accordingly, this systematic review was to detect and integrate techniques of reinforcing and maintaining human resources in long-term crises in healthcare organizations.

## 2. Materials and Methods

The paper is presented in accordance with the Preferred Reporting Items for Systematic Reviews (PRISMA) reporting checklist (Supplementary 1).

### 2.1. Search Strategy

A comprehensive search was first conducted on PubMed, Scopus, Web of Science, and Cochrane databases from 2003 due to the occurrence of the first long-term crisis (SARS) to April 2021 to detect the best techniques to maintain human resources of the health system during crises and under stressful conditions. To this end, the following keywords, either alone or by conjunctions of “and” or “or,” were used to find relevant papers with the concerned keywords in the title, abstract, and keywords sections: “Management,” “Strategy,” “Method,” “Support,” “Maintenance,” “Sustaining,” “Compliance,” “Human Resources,” “Healthcare worker,” “Physicians,” “Doctors,” “Nurses,” “Medical staff,” “Crisis,” “Disaster,” “Catastrophic,” “Systematic review,” Long term,” and “Long-term.” We manually searched the reference lists to identify studies missed. In addition, we contacted with authors included articles for identifying more studies.

### 2.2. Study Selection

Inclusion criteria were systematic reviews in English, full-text papers, and healthcare workers as the statistical population. Exclusion criteria were nonsystematic reviews, irrelevant studies, qualitative studies, studies presented in conferences and/or seminars, and letters to the editors.

**Figure 1:**
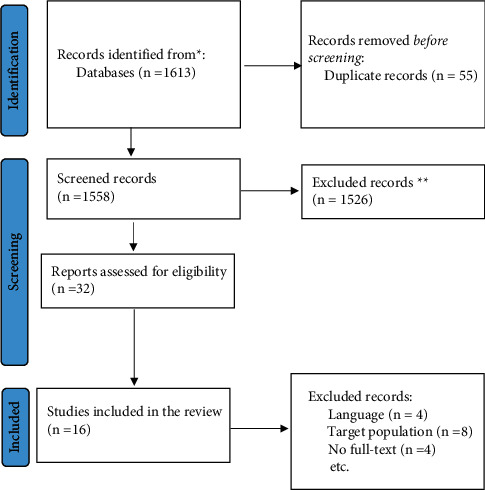
Reporting items for systematic reviews flowchart of the included studies.

To select the articles and extract the data, initial screening was based on titles and abstracts. The articles were evaluated independently. The abstracts lacking data were revised for full-text assessment. Then two researchers individually evaluated the full text of the articles and determined their fitness (Figure 1).

### 2.3. Data Extraction and Quality Assessment

Two researchers (A. S. and M. M.) extracted data regarding standard criteria, and the results were reviewed by two senior researchers (M. K. and S. K.). The extracted data included the first author, year of publication, period, objectives, number of studies, and findings. The studies were evaluated using a checklist entitled “Measurement Tool to Assess Systematic Reviews (AMSTAR)” [11] to assess methodological quality (Supplementary 2). The tool consists of 11 items and has good face and content validity for measuring the methodological quality of systematic reviews.

## 3. Results

Based on Table 1, findings are classified in three areas of “organizational resilience,” “motivation-hygiene measures,” and “managers' soft skills.” These factors can help healthcare workers to resist and keep on in long-term crises.

### 3.1. Organizational Resilience

We categorized the organizational factors such as material resources, scarce resource allocation management strategies, preparation, planning, diverse programming for critical episodes, crisis treatment standards, critical incident stress management, risk management, social media, government's measures, leadership methods, organizational culture, special training program, education systems, information management, cooperation, and peer support in a set and according to the literature labeled it as “organizational resilience.” According to the standards, organizational resilience is the organization's ability to predict, plan, react, and adapt to crisis in order to survive and succeed [27, 28]. The organizational resilience has a significantly positive relation to the organizational members' intentions for contributing to organizational effectiveness after a crisis situation [29].

### 3.2. Motivators and Hygiene Factors

The human resource features factors are thought to be relevant to the maintenance and motivation factors to respond to the crisis. These are organized into motivators and hygiene factors category. Hygiene or maintenance factors do not motivate employees; however, they merely prevent dissatisfaction and maintain the status [30]. In our study, hygiene factors covered aspects such as providing safety and protective equipment, availability of resources, shift flexibility, rest, workload, financial awards, and job promotion. Motivation factors were inherently job-related and, in this study, motivators reflect the pursuing professional education, role transparency, footprint of the organization's risk, and adaptive capacity. Managers should be aware that satisfaction is associated with motivational factors and that dissatisfaction is associated with hygiene factors.

### 3.3. Managers' Soft Skills

“Soft skills” refer to all the competencies that are not directly related to a specific task. The soft skills enable managers to succeed in an increasingly complex, interconnected, and rapidly changing situation such as crisis. The social abilities, language and communication capability, and friendliness and team working ability, as well as other personality traits that characterize relationship between people are soft skills [31]. So, listening skills, emotional skills, empathy, complex problem-solving, creative strategies to support and manage personnel, critical thinking, learning to learn, collaboration, creating opportunities for development for the employees working under crisis conditions, and generally sparing efforts to build trust were classified in the managers' soft skills category.

## 4. Discussion

In this study, some review studies on the techniques causing the reinforcement and maintenance of human healthcare resources under the long-term crisis conditions were examined, according to which three main dimensions were detected.

The first dimension was organizational resilience. Resilience is defined as an organization's capability to continue its activities in line with its goals in the presence of challenges. Resilience not only increases the system's capacity to resist strikes but also adapts and changes in accordance with the challenges. In this regard, managers need to consider crises in advance and adopt some management techniques such as the accelerated successive overrelaxation and ASOR method, identify the organization's weaknesses and strengths, and make necessary plans [9]. This planning is also necessary for the provision of human resources. In crises, workers could be classified into three groups. The first group includes workers who work in the organization and continue their activities during the crises period. This group of workers might either be satisfied with their jobs or not be satisfied and stay in the organization due to their lack of willingness to search for a new job and family commitments, while they are dissatisfied with their jobs. This is not good for the organization since such workers fail to be creative and dynamic and finally quit the organization when they find an appropriate job. Organization managers must take management measures to promote the satisfaction of dissatisfied and unmotivated workers because the lack of motivation could decrease the quality of service [11,32]. The second group includes workers lost because of job dissatisfaction, retirement, death, or other causes [32]. The third group encompasses workers coming to the organization during the crisis. These workers might be just graduated or senior students, who join the organization with the incentive of employment upon the call of the health system [32]. According to Collado-Boira, despite the threats of COVID-19, senior students were more willing to accept the government's request due to their social commitment and professional ethics [11].

In the three groups, job satisfaction is considered a critical factor in maintaining staff under crisis conditions. The other finding of this study was the necessity of motivation-hygiene measures, which are the activities performed by health managers and policymakers for their staff to help them maintain their morals. These measurements positively affect the organization's output, thus providing high-quality care since these outputs are the results of the staff's efforts.

Moreover, the motivation-hygiene measures decrease the likelihood of staff leaving service and changing job, thereby decreasing the organization's costs [33]. Factors such as job security, flexible attendance time, and participation in decision-making increase the staff's willingness to work and, consequently, their maintenance in the organization [34]. Furthermore, Pavani Rangachari's investigations in 2017 indicated that excluding nurses from the decision-making process was the leading cause of job burnout [33]. According to Chia-Ching Wu's research findings on nurses' willingness to care for patients with SARS in 2012, the workers' willingness to work during crisis periods does not change significantly, and the majority of workers are willing or unwilling under normal conditions. In this investigation, considering their financial needs, family support, safeguarding individual health, and human resource policies, nurses found it a better decision to stay in their jobs during the outbreak of SARS [24]. Therefore, motivation-hygiene measures need to be adopted under both crisis and normal condition [13–15] since workers' reluctance to work in both conditions reduces the quality of work and service even if it does not make workers quit their jobs. This will be more alarming under the crisis condition.

Nevertheless, Mary Chaffee's findings are in contrast with those reported by Chia-Ching Wu. Considering 30 studies, she stated that biological events might significantly decrease individuals' willingness to work; however, the provision of protective and training equipment is likely to improve their willingness [35]. According to the critical role of motivation-hygiene measures in establishing trust, they are of great importance in promoting job satisfaction and willingness to work, and precise economic planning should be proposed to provide their financial resources [36,37]. To manage crisis and achieve the best results under the crisis, the measures can be categorized according to their priority. According to Herzberg's motivation-hygiene theory, hygienic factors such as security, work, and salary decrease dissatisfaction; however, they cannot enhance satisfaction. This is while motivational factors promote job satisfaction. According to workers' needs, an increase in motivation-hygiene measures can play a pivotal role in maintaining workers in the long-term crises. These measures in healthcare systems include the provision of protective equipment required by workers, shift change programs to let the worker rest [12], information dissemination [24], training programs, and increased awareness in dealing with illness, all of which would decrease their feelings of fear [13]. High work pressure should also be considered since it has negative effects on the nurses' performance. In this regard, if a nurse's mistake aroused by high work pressure under a crisis condition makes a patient die or get worse, the consequences have destructive effects on the nurse's morals and her/his willingness to continue working [38]. Accordingly, employee new graduates and entrusting simple tasks to these individuals would make more experienced health workers have more time and concentrate on handling high-level care services [3]. Moreover, if it is possible not to reduce salaries with a decrease in work hours, a motivation-hygiene measure should be adopted, which would have a positive effect during the crisis period [39]. The provision of personal protective equipment can significantly influence individuals' mental and physical health. It makes workers be less concerned with their health status and their families' health, as such the likelihood of disease transmission is decreased [33, 38]. Moreover, training, public health, families' awareness of mental and social supports, and the appreciation of workers are considered motivation-hygiene measurements that positively influence workers' mental and physical health and prevent job burnout and the loss of healthcare workers under such conditions [15].

One of the main study findings was the necessity of developing managers' soft skills. The skills are as significant as establishing an intimate relationship with workers to promote organizational resilience in crisis. Organizations with more competent managers respond to crises better [14, 40]. Establishing effective relationship under critical conditions promotes trust among workers [33]. Trust as the driving force of ethics and the incentive for employees is of great importance for organizations, especially in crisis since psychological trust and safety are the prerequisites of organizational resilience in health organizations [33, 41]. Such measures require not many financial resources; however, managers' attention and managerial skills are of paramount importance. The measures include tasks such as listening to workers, learning from their personal problem-solving experiences, making clear strategies to support and manage workers, and generally sparing efforts to build trust.

Moreover, given that these measures can create a sense of trust in employees, providing opportunities to promote employees in crises had positive effects on the maintenance of workers in the organization in crisis [42]. The sense of trust and mental safety among healthcare workers is necessary to let them convey their concerns to managers under such conditions since managers can eliminate the leading causes of mistakes and hinder their recurrence [33]. According to the Social Exchange Theory (SET), when employees find the organization a supportive environment, where their social-emotional needs are met and favorable working conditions are provided, a psychological attachment is created, making them committed to the organization [43].

In general, flexibility in strategic planning and human resource planning and the provision of motivation-hygiene measures for employees can improve the likelihood of survival for health workers under critical conditions. This comes true, especially if these measures are accompanied with promoting managers' soft skills to establish an effective relationship having significant positive effects on the maintenance of individuals and improving their performance in the organization [42].

The strengths of our study include the use of appropriate databases in identifying potentially eligible review studies, providing search strategy in detail, selecting the studies based on AMSTAR, and reporting based on a PRISMA 2020 checklist. The limitations of our study may be due to the use of systematic reviews with no registration predetermined protocol. Although we searched the Cochrane database for unplanned duplication, the study protocol was only registered at the Student Research Committee after the Research Ethics Committee confirming to enable the representative of the Research Vice-Chancellor at Hamadan University of Medical Sciences in comparing the reported review with what was planned in the protocol.

## 5. Conclusion

In the present review study, three dimensions (namely, resilience, motivation-hygiene measures, and development of managers' soft skills) are identified as the main factors maintaining employees in healthcare centers in long-term crises. Incentives and motivational measures promote job satisfaction among employees and consequently increase their willingness to work and stay in a concerned organization. Under crisis, only motivational measures are not enough to maintain employees in the organization, and managers need to be equipped with strong soft skills as communication skills to meet the employees' physical and psychological needs and build trust among them. Some further plans and programs should be put forth to be performed in the best way to maintain workers in organizations.

## Figures and Tables

**Table 1:** Summaries of the main points of the article.

Name of the first author	Year	Period	Objectives	Databases	Number of reviewed articles	Meta	Findings	Subset
Muller et al. [12]	2020	01.12.2019–11.05.2020	1. Investigating the impact of the COVID-19 pandemic on healthcare workers' mental health, including (a) changes over time, (b) prevalence of mental health problems and risk/resilience factors, (c) strategies and resources used by healthcare providers to protect one's mental health, (d) perceived need and preferences for interventions, and (e) healthcare workers' understandings of their mental health during the pandemic 2. Describing the interventions assessed in the literature to prevent or reduce negative impacts on the mental health of healthcare workers working during the COVID-19 pandemic	PubMed, CDC, EMBASE, and NIPH's	59	N	1. Healthcare staff in different positions are exposed to risks such as depression, anxiety, and sleep problems during the COVID-19 pandemic due to different reasons 2. Preparing appropriate personal protective equipment and shift change programs to allow for rest in long-term crises is important	Motivation-hygiene measures

Cabarkapa et al. [13]	2020	2002–21.08.2020	1. Investigating psychological disorders in healthcare workers (HCV) during severe epidemics 2. Identifying strategies to solve problems	PubMed, MEDLINE, and CINAHL	55	N	1. Psychological consequences in healthcare staff, such as the risk of problems and anxiety, are different. The fear of unknown cases is one of their challenges 2. The negative mentality created by family and society increases the negative effects of stress 3. Adaptative methods are different among different cultures, physicians, nurses, and other healthcare employees	Motivation-hygiene measures

Willis-Shattuck et al. [14]	2008	1980–09.2007	1. Identifying factors affecting motivation 2. Investigating the effectiveness of interventions in improving motivation and reducing healthcare workers' immigration in developing countries	PubMed, ISI Web of Science, EMBASE/Medline, and Google Scholar	20	N	The following seven motivational factors contribute to maintaining workers: 1. Financial awards 2. Job promotion 3. Pursuing education 4. Hospital infrastructure 5. Availability of resources 6. Hospital management 7. Appreciation	Motivation-hygiene measures Managers' soft skills

Barasa [9]	2018	Till 31.12.2016	1. Exploring how resilience is conceptualized 2. Identifying factors affecting organizational resilience and how they are nurtured	PubMed, Econlit, EBSCOHOST databases, Google, and Google Scholar	23	N	Organizational resilience is affected by material resources, preparation, and planning, information management, sideways of increasing revenue, government's measures, leadership methods, organizational culture, human capital, social media, and cooperation	Organizational resilience

Fernandez et al. [15]	2020	Till 03.2020	Identifying factors affecting the provision of high-quality services by nurses during the epidemic	CINAHL, MEDLINE, EMBASE, PubMed, Google Scholar, Cochrane Library, MedNar, and ProQuest	28	N	The nursing team provide high-quality care if the following factors are available: 1. Physical and emotional supports 2. Organization and management's timely response and reaction	Managers' soft skills

Anderson et al. [16]	2020	2008–9.12.2019	Investigating the effectiveness of organizational peer support and crisis-focused psychological interventions to mitigate posttraumatic stress injuries (PTSIs) among public safety personnel (PSP), frontline healthcare professionals (FHP), and other relevant groups at risk of occupational exposure to potentially psychologically traumatic events (PPTEs)	PsycINFO, PubMed, JSTOR, Web of Science, Wiley, Sage, Taylor & Francis, Cambridge and Oxford Journal Online, Google Scholar	14	N	Public safety personnel (PSP) and frontline healthcare professionals (FHP) are often exposed to potentially psychologically traumatic events (PPTEs), and the increase in the rates of posttraumatic stress injuries (PTSIs) such as frequent absences, labeling, smoking and drugs, and suicide are also noticed among them Measures such as diverse programming for critical incident stress debriefing, critical incident stress management, peer support, psychological first aid, and trauma risk management result in the reduction of PTSI	Organizational resilience, Managers' soft skills

Aoyagi et al. [17]	2015	04.2013–2015	1. Investigating willingness to work in HCWs during the flu pandemic 2. Identifying factors affecting the willingness to work	MEDLINE, EMBASE, Web of Knowledge, SCOPUS, AMED, ASSIA, BioEthicsWeb, CINAHL, Cochrane Library, PsychINFO, Google Scholar, and OpenGrey	43	Y	The following factors were associated with an increase in the willingness to work in healthcare workers, physicians, and nurses: 1. Permanent employment 2. Perceived personal safety 3. Awareness of the pandemic risk and clinical knowledge of epidemics 5. Teaching how to respond to the epidemic 6. Self-confidence in personal skills. However, childcare was significantly associated with a reduced willingness to work during the flu pandemic.	Motivation-hygiene measures Managers' soft skills

Ashcroft et al. [18]	2020	1996–19.03.2020	1. Reviewing disaster training courses for medical students systematically 2. Describing the educational structure and methodology 3. Evaluating both preparedness for disaster medicine and learning outcomes to inform the development of COVID-19-specific training programs	EMBASE, Medline, and Cochrane	23	N	To attract medical students aimed at helping dealing with COVID-19, there is a need for a special training program for them. This study indicates that medical students trained by appropriate education systems can play a pivotal role in managing the epidemic	Managers' soft skills Organizational resilience

Raphael et al. [19]	2021	Till 30.04.2021	Investigating the adaptability of providing mental health services during the outbreak of COVID-19	CINAHL, EMBASE, Medline, PsycINFO, and Web of Science	19	N	Mental health services need to consider infection control measures and implement service changes to support continuity of care and patient and staff wellbeing. Services also need to ensure they communicate important information in a clear and accessible manner with their staff and patients regarding service delivery, contagion symptoms, government guidelines, and wellbeing	Managers' soft skills

Pappa et al. [20]	2020	Till 17.04.2020	Evaluating the prevalence of depression, anxiety, or insomnia	MEDLINE, PubMed, and Google Scholar	13	Y	Compared with men and medical staff, women and nurses show more emotional symptoms associated with anxiety and depression. Special arrangements such as the provision of protective equipment for staff and their family should be made to protect their mental health.	Motivation-hygiene measures

Gross et al. [21]	2021	Till 26.04.2020	1. Examining HCV's physical and mental health status in the COVID-19 situation 2. Detecting corona risk prevention measures	PsycINFO, Web of Science, and PubMed	27	N	Because of the high exposure of healthcare staff to COVID-19, it is necessary to 1. make the workplace safe 2. increase the healthcare staff information level of how infections outbreak and affect health	Motivation-hygiene measures

Bhaumik et al. [22]	2020	2020	Identifying CHWS' principal roles, issues, obstacles, and activators to respond to the pandemic	PubMed, 18 websites of different government ministries (India, Australia, and Singapore), public health agencies (from China, US, South Africa, UK, Hong Kong, and Australia), multinational agencies (WHO, European CDC, and African CDC), COVID-19 resource aggregators available at the time of review (Wiley, Elsevier, Oxford University Press, New England Journal of Medicine, Journal of the American Medical Association), and preprints (medRxiv)	36	N	Healthcare workers play a pivotal role in pandemics. In addition to job satisfaction and workers' health, it is necessary to ensure role transparency, education, support, and supervision	Managers' soft skills Motivation-hygiene measures

Pollock et al. [23]	2020	2002–2020	1. Evaluating the intervention effects aimed at supporting the frontline healthcare professionals' resilience and mental health during the outbreak of disease and epidemics 2. Identifying obstacles and facilitators effective in performing interventions aimed at supporting frontline healthcare professionals' resilience and mental health during the outbreak of disease and epidemics	CENTRAL, MEDLINE, EMBASE, Web of Science, PsycINFO, CINAHL, Global Index Medicus databases, and WHO	16	N	Frontline healthcare professionals' mental health and resilience during epidemics can be improved by workplace interventions, supportive interventions to meet basic daily needs, psychopathic interventions, health interventions, or a combination of these.	Motivation-hygiene measures Managers' soft skills

Timbie et al. [24]	2013	1990–2011	The analysis of scarce resource allocation management strategies	Medline, Scopus, EMBASE, Cumulative Index to Nursing and Allied health literature, Global Health, Web of Science, Cochrane, New York Academy of Medicine's Grey literature report, and key websites	74	N	Scarce resource allocation management strategies during collective explosion events include 1. reducing demand for healthcare services 2. optimizing the use of available resources 3. increasing existing resources 4. implementing crisis treatment standards and hybrid strategies	Organizational resilience

Allan et al. [25]	2020	Till 30.03.2020	Examining HCWs' mental health disorders regarding the increasing rate of hospital admissions and mortality due to COVID-19	Medline, PsycINFO, CINAHL, PubMed, OVID, and ScienceDirect	19	Y	Mental health disorders are common among healthcare workers in contact with patients during the epidemic, but the trend of these disorders has not been completely understood. More followup of healthcare workers is necessary.	Managers' soft skills

De Brier et al. [26]	2020	Till 28.03.2021	Identifying risk and protective factors of HCW's mental health during the corona epidemic	MEDLINE, EMBASE, PsycINFO, WHO, and CDC	33	N	There are some pieces of evidence indicating that clear communication, organizational support, social support, and a sense of personal control are protective factors against mental consequences.	Managers' soft skills

## Data Availability

The data are available by contact with the corresponding author.

## Supplementary Materials

Supplementary Materials Supplementary 1: the PRISMA checklist. The Preferred Reporting Items for Systematic Reviews and Meta-Analysis (PRISMA) is a 27-item checklist used to improve transparency in systematic reviews. These items cover all aspects of the manuscript, including title, abstract, introduction, methods, results, discussion, and funding. Supplementary 2: evaluating the quality of articles using AMSTAR checklist. The included articles were evaluated using a checklist entitled “Measurement Tool to Assess Systematic Reviews (AMSTAR)” [11] to assess methodological quality. The tool consists of 11 items and has good face and content validity for measuring the methodological quality of systematic reviews.
