# Supplementary material for: Reinforcement and Maintenance of Human Resources for Health Systems during Long-Term Crises: A Systematic Review of Systematic Reviews
Source: Emerg Med Int. 2021 Oct 31;2021:9613443. doi: 10.1155/2021/9613443 (PMC8572622; doi:10.1155/2021/9613443)
Supplement: Supplementary Materials — Supplementary 1: the PRISMA checklist. The Preferred Reporting Items for Systematic Reviews and Meta-Analysis (PRISMA) is a 27-item checklist used to improve transparency in systematic reviews. These items cover all aspects of the manuscript, including title, abstract, introduction, methods, results, discussion, and funding. Supplementary 2: evaluating the quality of articles using AMSTAR checklist. The included articles were evaluated using a checklist entitled “Measurement Tool to Assess Systematic Reviews (AMSTAR)” [11] to assess methodological quality. The tool consists of 11 items and has good face and content validity for measuring the methodological quality of systematic reviews. [file 9613443.f1.zip › 9613443.f1/Supplementary2.docx]

Table 1 Table 1 Evaluate the quality of articles using AMSTAR checklist

|  | | AMSTAR score | | | | | | | | | | | | AMSTAR score  Summary | | | |  |
| --- | --- | --- | --- | --- | --- | --- | --- | --- | --- | --- | --- | --- | --- | --- | --- | --- | --- | --- |
| Author | **Reference** | Was an ‘‘a priori’’ design provided? | Was there duplicate study selection and data extraction? | Was a comprehensive literature search performed? | Was the status of publication (i.e., grey literature) used as an inclusion criterion? | Was a list of studies (included and excluded) provided? | . Were the characteristics of the included studies provided? | Was the scientific quality of the included studies assessed and documented? | Was the scientific quality of the included studies used appropriately in formulating conclusions? | | Were the methods used to combine the findings of studies appropriate? | Was the likelihood of publication bias assessed? | Was the conflict of interest included? | YES | NO | Can’t answer | Not applicable | |
| Ashley Elizabeth Muller | 18 | NO | YES | YES | NO | YES | NO | YES | | YES | Not applicable | NO | YES | 6 | 4 | 0 | 1 | |
| Sonja Cabarkapa | 19 | NO | YES | YES | Cant answer | YES | YES | YES | | YES | Not applicable | NO | YES | 7 | 2 | 1 | 1 | |
| Mischa willis-shattuck | 20 | NO | YES | YES | YES | YES | YES | YES | | Cant answer | Not applicable | NO | YES | 7 | 2 | 1 | 1 | |
| Edwine Barasa | 14 | NO | YES | YES | YES | YES | Not applicable | YES | | YES | Not applicable | NO | YES | 7 | 2 | 2 | 0 | |
| Ritin Fernandez | 21 | YES | YES | YES | YES | YES | YES | YES | | YES | Not applicable | YES | YES | 10 | 0 | 0 | 1 | |
| Gregory S. Anderson | 22 | YES | YES | YES | NO | YES | YES | YES | | Cant answer | Not applicable | YES | YES | 8 | 1 | 1 | 1 | |
| Yumiko Aoyagi | 23 | YES | YES | YES | YES | YES | YES | YES | | NO | YES | YES | YES | 10 | 1 | 0 | 0 | |
| James Ashcroft | 24 | YES | YES | YES | YES | YES | YES | YES | | YES | Not applicable | YES | NO | 9 | 1 | 0 | 1 | |
| Jessica Raphael | 25 | NO | NO | YES | YES | YES | NO | YES | | YES | Not applicable | YES | NO | 6 | 4 | 0 | 1 | |
| Sofia Pappa | 26 | NO | YES | NO | NO | YES | YES | YES | | YES | Not applicable | YES | NO | 6 | 4 | 0 | 1 | |
| J Valerie Gross | 27 | NO | YES | YES | YES | YES | NO | YES | | NO | YES | NO | YES | 7 | 4 | 0 | 0 | |
| Soumyadeep Bhaumik(28) | 28 | NO | YES | YES | YES | NO | YES | YES | | Cant answer | Not applicable | NO | YES | 6 | 3 | 1 | 1 | |
| Pollock A | 29 | YES | YES | YES | YES | YES | NO | YES | | YES | Not applicable | NO | NO | 7 | 3 | 0 | 1 | |
| JW Timbiea | 30 | YES | YES | YES | YES | YES | YES | YES | | NO | Not applicable | NO | NO | 7 | 3 | 0 | 1 | |
| Sophie M. Allan | 31 | NO | YES | YES | YES | YES | YES | YES | | Cant answer | YES | YES | YES | 9 | 1 | 1 | 0 | |
| Niels De Brie | 32 | YES | YES | YES | YES | YES | YES | NO | | YES | Not applicable | NO | YES | 8 | 2 | 0 | 1 | |

References:

14. Barasa E, Mbau R, Gilson L. What is resilience and how can it be nurtured? A systematic review of empirical literature on organizational resilience. International journal of health policy and management. 2018;7(6):491.

18. Muller RAE, Stensland RSØ, van de Velde RS. The mental health impact of the covid-19 pandemic on healthcare workers, and interventions to help them: A rapid systematic review. Psychiatry research. 2020:113441.

19. Cabarkapa S, Nadjidai SE, Murgier J, Ng CH. The psychological impact of COVID-19 and other viral epidemics on frontline healthcare workers and ways to address it: A rapid systematic review. Brain, behavior, & immunity-health. 2020:100144.

20. Willis-Shattuck M, Bidwell P, Thomas S, Wyness L, Blaauw D, Ditlopo P. Motivation and retention of health workers in developing countries: a systematic review. BMC health services research. 2008;8(1):1-8.

21. Fernandez R, Lord H, Halcomb E, Moxham L, Middleton R, Alananzeh I, et al. Implications for COVID-19: a systematic review of nurses’ experiences of working in acute care hospital settings during a respiratory pandemic. International Journal of Nursing Studies. 2020:103637.

22. Anderson GS, Di Nota PM, Groll D, Carleton RN. Peer support and crisis-focused psychological interventions designed to mitigate post-traumatic stress injuries among public safety and frontline healthcare personnel: a systematic review. International journal of environmental research and public health. 2020;17(20):7645.

23. Aoyagi Y, Beck CR, Dingwall R, Nguyen‐Van‐Tam JS. Healthcare workers' willingness to work during an influenza pandemic: A systematic review and meta‐analysis. Influenza and other respiratory viruses. 2015;9(3):120-30.

24. Ashcroft J, Byrne MH, Brennan PA, Davies RJ. Preparing medical students for a pandemic: a systematic review of student disaster training programmes. Postgraduate Medical Journal. 2020.

25. Raphael J, Winter R, Berry K. Adapting practice in mental healthcare settings during the COVID-19 pandemic and other contagions: systematic review. BJPsych Open. 2021;7(2).

26. Pappa S, Ntella V, Giannakas T, Giannakoulis VG, Papoutsi E, Katsaounou P. Prevalence of depression, anxiety, and insomnia among healthcare workers during the COVID-19 pandemic: A systematic review and meta-analysis. Brain, behavior, and immunity. 2020.

27. Gross JV, Mohren J, Erren TC. COVID-19 and healthcare workers: a rapid systematic review into risks and preventive measures. BMJ open. 2021;11(1):e042270.

28. Bhaumik S, Moola S, Tyagi J, Nambiar D, Kakoti M. Community health workers for pandemic response: a rapid evidence synthesis. BMJ global health. 2020;5(6):e002769.

29. Pollock A, Campbell P, Cheyne J, Cowie J, Davis B, McCallum J, et al. Interventions to support the resilience and mental health of frontline health and social care professionals during and after a disease outbreak, epidemic or pandemic: a mixed methods systematic review. Cochrane Database of Systematic Reviews. 2020(11).

30. Timbie JW, Ringel JS, Fox DS, Pillemer F, Waxman DA, Moore M, et al. Systematic review of strategies to manage and allocate scarce resources during mass casualty events. Annals of emergency medicine. 2013;61(6):677-89. e101.

31. Allan SM, Bealey R, Birch J, Cushing T, Parke S, Sergi G, et al. The prevalence of common and stress-related mental health disorders in healthcare workers based in pandemic-affected hospitals: a rapid systematic review and meta-analysis. European journal of psychotraumatology. 2020;11(1):1810903.

32. De Brier N, Stroobants S, Vandekerckhove P, De Buck E. Factors affecting mental health of health care workers during coronavirus disease outbreaks (SARS, MERS & COVID-19): A rapid systematic review. PloS one. 2020;15(12):e0244052
